# A Comprehensive Spectroscopic Analysis of the Ibuprofen Binding with Human Serum Albumin, Part I

**DOI:** 10.3390/ph13090205

**Published:** 2020-08-21

**Authors:** Anna Ploch-Jankowska, Danuta Pentak

**Affiliations:** 1 Faculty of Pharmaceutical Sciences in Sosnowiec, Medical University of Silesia in Katowice, Doctoral School, Jedności 8, 41-200 Sosnowiec, Poland; 2 Faculty of Chemistry, University of Opole, Oleska 48, 45-052 Opole, Poland; danutapentak@poczta.onet.pl

**Keywords:** ibuprofen, human serum albumin, spectrophotometric, spectrofluorometric analyses

## Abstract

Human serum albumin (HSA) plays a fundamental role in the human body. It takes part in the transport of exogenic and endogenic substances, especially drugs. Ibuprofen (IBU) is one of the most commonly used non-steroidal anti-inflammatory drugs, used for pain relief, fever relief, and for anti-inflammatory purposes. The binding of ligands with HSA is a significant factor which determines the toxicity and the therapeutic dosages of these substances. The aim of this study was to compare the degree of ibuprofen binding with human serum albumin at various temperatures and protein solution pH values. In order to evaluate conformational changes in HSA caused by interaction with ibuprofen, spectrophotometric (first and second derivatives of the UV-VIS spectrum), and spectrofluorometric analyses were performed concerning the mutual interactions of IBU-HSA. The use of fluorescent spectroscopy allowed for recording fluorescent emissive spectra of HSA (5 × 10^−6^ mol/dm^3^) without and with the presence of ibuprofen (1 × 10^−5^–1 × 10^−4^ mol/dm^3^) at temperatures of 308, 310, 312, and 314 K at pH values of 6.5, 6.8, 7.4, 7.8, and 8.1. System fluorescence was excited by radiation of wavelengths of λex = 275 nm and λex = 295 nm. Based on this, original and modified Stern-Volmer, Scatchard, Klotz and Hill curves were determined. The data that were obtained showed a significant effect of temperature and pH of the human serum albumin solution on the strength and type of interaction of ibuprofen with HSA.

## 1. Introduction

Human serum albumin (HSA) is the most important blood serum protein, consisting of a single polypeptide chain of 585 amino acids [1,2,3,4]. Crystallographic structure analysis has shown that HSA consists of three structural domains (I–III). Each of these is made up of subdomains A and B [3,4]. Its tertiary structure consists of 17 disulfide bridges, one free thiol group from a cysteine residue (Cys-34), one tryptophanyl residue (Trp-214), and 17 tyrosyl residues [1,2,3,4,5,6]. Studies performed by Sudlow and Carter have proven the existence of specific sites in the HSA structure that bind to drugs—site I, called the warfarin binding site, site II also called the benzodiazepine binding site as well as six ligand binding sites, which differ with respect to their structure [3,7,8]. It has also been proven that drug binding sites I and II according to Sudlow correlate with subdomains IIA and IIIA [9]. Human serum albumin plays a key role in the human body. It is responsible for maintaining 75–80% of the osmotic pressure value, and for ensuring correct blood pH. It has the ability to bind and transport many exo- and endogenic substances, e.g., drugs, fatty acids, vitamins, metabolites, dyes, hormones, amino acids, and metal ions [3,4,6]. It is commonly known that the strength of a drug’s effect depends above all on its ability to bind with the protein. It has been proven that serum pH, temperature, physicochemical properties of the ligand, its affinity to binding sites, the protein concentration as well as the presence of other exo- and endogenic substances has an effect on the effectiveness of drug compounds [10,11,12].

In this work, an analysis was performed of the conformational changes of human serum albumin caused by interaction with ibuprofen. The study was performed at temperatures of 308, 310, 312, and 314 K and pH values of the protein solution of 6.5, 6.8, 7.4, 7.8, and 8.1. The study was performed with the aid of such spectroscopic techniques as spectrophotometry UV-VIS and spectrofluorometry.

**Figure 1:**
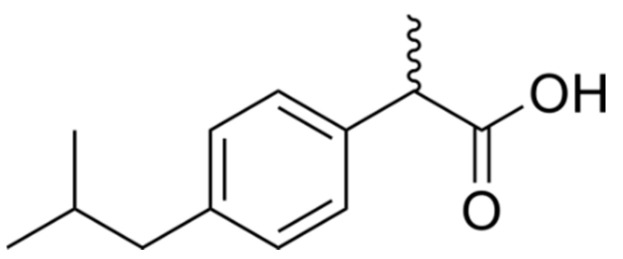
Chemical structure of ibuprofen (IBU).

Ibuprofen ((RS)-2-(4-(2-methylpropyl) phenyl) propanoic acid, IBU, Figure 1) is a non-steroidal anti-inflammatory drug used for treating pain, fever, and inflammation [13].

**Figure 2:**
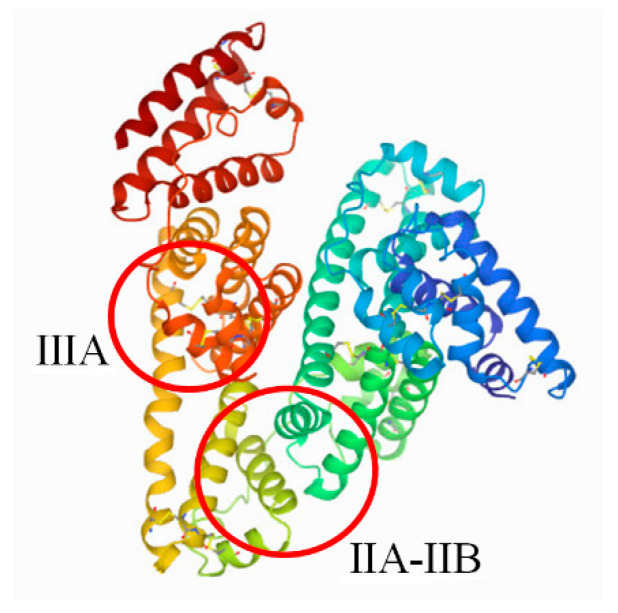
The main ibuprofen binding sites in the human serum albumin (HSA) molecule (PDB ID: 1AO6) [18,19].

It is often applied in cases of painful menstruation and tooth, head, muscle, and post-traumatic pain. IBU is used for reducing fever, for relieving symptoms of the common cold and flu, and for treating rheumatoid arthritis and osteomyelitis [14,15]. Ibuprofen shows a very high affinity for human serum albumin, exhibiting a high affinity for plasma proteins (90–99%) [16]. The major binding sites for ibuprofen in the human serum albumin are found in subdomain IIA, IIB, and IIIA (Figure 2) [17].

Despite the known binding sites of ibuprofen in the human serum albumin molecule, in the literature there is no information on Stern-Volmer constant K_SV_ values, association constants K_a_, number of binding sites (*n*) and the Hill’s coefficient (*n_H_*) in the analyzed temperature range, buffer pH, human serum albumin, and ibuprofen concentration. Ibuprofen is a nonselective cyclooxygenase enzymes (COX) inhibitor. It takes part in inhibiting two isoforms of cyclooxygenase: The cyclooxygenase enzymes COX-1 and COX-2 [20].

Human serum albumin performs a buffering function, maintaining a constant blood pH. The physiological pH value of serum which ensures correct human body function is 7.35–7.45. The pH boundary values are within the ranges of 6.80–7.35 and 7.45–7.80 [21]. Changes in pH values may cause structural changes in HSA.

In order to analyze the effect of temperature on ibuprofen binding with human serum albumin, a study was performed concerning blood-protein interaction in the temperature range of 308–314 K, because this temperature corresponds to physiological conditions (T = 310 K), a state of lowered body temperature (T = 308 K, temperature close to the extreme value of therapeutic hypothermia used in clinical conditions 307 K [22]) as well as states of inflammation (T = 312 K and T = 314 K) in the human body.

We selected ibuprofen for this study, despite other NSAIDs because ibuprofen is the most commonly used and frequently prescribed nonsteroidal anti-inflammatory drug [16]. Ibuprofen near aspirin and naproxen is available over the counter (OTC) in most countries [23]. Moreover, this study performed in Ireland by Frank Bradbury involving more than 9000 patients, demonstrated that diclofenac, nimesulide, and ibuprofen were most frequently prescribed by general practitioners in this country—80% of total prescriptions [24].

## 2. Results and Discussion

Spectroscopic analysis of ibuprofen with human serum albumin interaction.

### 2.1. The Effect of Temperature on Human Serum Albumin Binding Properties

The absorption spectroscopy technique (UV-VIS) may be effectively utilized for analyzing the structural changes of blood serum albumin and for studying the protein-ligand complex. The human serum albumin UV-VIS spectrum in the wavelength range of 250–305 nm encompasses the absorption spectrum of aromatic amino acids (Trp, Tyr, Phe) [25,26].

The first derivative of the UV-VIS spectrum, expressed as dA/dλ, determines the absorption speed (A), which changes with the changing wavelength (λ). The second derivative d^2^A/d^2^λ describes the speed of this change. Literature suggests that subtle changes in the protein tertiary structure, which are faintly visible on the zero-order absorption spectrum, become clear on the spectrum second derivative [27,28]. The spectrum second derivative distinguishes three wavelength ranges. The spectral range from 250–270 nm encompasses the phenylalanine residue (Phe). The effect of other aromatic chromophores in the wavelength range is negligible. The wavelength range of 293–305 nm describes only the tryptophanyl residue (Trp), while the range of 270–293 nm encompasses the tyrosyl residues (Tyr) and tryptophanyl residue in HSA [29,30].

The UV-VIS human serum albumin spectra (first and second derivative) at various temperatures are shown in Supplementary Figure S1.

Based on these studies (Supplementary Figure S1) it has been found that at temperatures of 308 and 310 K the HSA absorbance values in the studied wavelength range (250–320 nm) are the same. An increase in the temperature by 4 and 6 K, respectively causes an increase in the absorbance values. By analyzing the second derivative, it was found that changes in temperature in the range of 308–314 K do not have an effect on the tertiary structure of blood serum albumin.

Based on the presented UV-VIS spectra with and without the presence of ibuprofen (Supplementary Figure S2) it can be concluded that at a temperatures of 308, 312, and 314 K ibuprofen does not significantly affect the human serum albumin structure from a spectrophotometric point of view. On the other hand, at a temperature of 310 K, the human serum albumin absorbance value at a wavelength of 280 increases significantly after the addition of ibuprofen. This confirms the interaction between the protein and the ligand. Based on the second derivative of HSA absorption spectra (Supplementary Figure S2), changes were also noticed between the spectrum of the pure protein and albumin bound with ibuprofen at a wavelength range of 293–298 nm. These subtle differences in the curves of HSA and IBU-HSA testify to the effect of ibuprofen on changes in the protein tertiary structure around the tryptophanyl residues.

In order to analyze the changes of the hydrophobic surrounding of the tryptophanyl and tyrosyl residues in human serum albumin in the presence of ibuprofen (1 × 10^−5^–1 × 10^−4^ M) within subdomains IIA, IIIA, IB, and IIB, a spectrofluorometric analysis was performed. The IBU-HSA system was excited by radiation with wavelengths of λex = 275 nm and λex = 295 nm. In HSA, we can distinguish two types of fluorophores—tyrosine and tryptophan. Radiation with a wavelength of λex = 275 nm excites both tryptophanyl residues (Trp-214 located in subdomain IIA) as well as tyrosyl group residues localized in subdomains IB (Tyr-138, Tyr-140, Tyr-148, Tyr-150, Tyr-161), IIB (Tyr-319, Tyr-332, Tyr-334, Tyr-341, Tyr-353, Tyr-370), IIA (Tyr-263), and IIIA (Tyr-401, Tyr-411, Tyr-452, Tyr-497) [31,32]. Radiation of λex = 295 nm wavelength excites only the protein tryptophanyl residue (Trp-214) [3].

Based on the emissive fluorescent spectra (Supplementary Figure S3), an increase in albumin fluorescence with increasing ibuprofen concentrations was found at both wavelengths at all studied temperatures. Graphs of the relationship of fluorophore fluorescence in HSA and the wavelength show a shift in the maximum of fluorescence emission in the direction of shorter wavelengths—blue shift (hypsochromic shift). This testifies to the increase in the hydrophobic character of the surrounding of the tryptophanyl and tyrosyl residues in subdomains IIA, IIIA, IB, and IIB [33].

**Figure 3:**
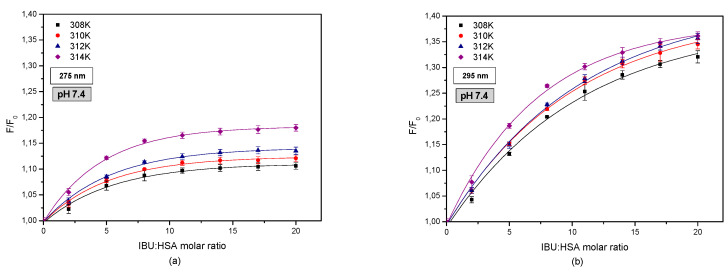
Fluorescence intensification/quenching curves of human serum albumin (5 × 10^−6^ M) at various concentrations of ibuprofen (1 × 10^−5^–1 × 10^−4^ M) in 308 K (■); 310 K (●); 312 K (▲); 314 K (◆), (**a**) λex = 275 nm, (**b**) λex = 295 nm, pH = 7.4.

Figure 3 shows fluorophore fluorescence quenching curves of HSA bound with IBU.

Analyzing the curves showing the relationship between HSA fluorescence $\left(F/F_{0}\right)$ and the molar ratio of IBU:HSA (Figure 3) an increase in the fluorophore fluorescence intensity was found in the entire analyzed concentration range. The changes that were observed concern the excitation of the IBU-HSA system by a wavelength of λex = 275 nm (Figure 3a) as well as λex = 295 nm (Figure 3b). A relationship was found between the fluorescence excitation strength and temperature only in the case of exciting tryptophanyl and tyrosyl HSA residues at a wavelength of λex = 275 nm and for ibuprofen concentrations below 4 × 10^−5^ M for the system excited by a wavelength of λex = 295 nm. Based on this analysis, it is suspected that by interacting with the protein molecule, ibuprofen breaks the tryptophan-tryptophan hydrogen bonds, which increases the number of free tryptophan molecules in the system. This in turn causes an increase in the tryptophanyl residue fluorescence. The change in the fluorescence band of the tryptophanyl residue confirms the interaction between tryptophan-ibuprofen and a free tryptophan molecule [34].

In order to verify whether tyrosyl residues (Tyr) play a role in the IBU-HSA bond, a differential spectrum was created for the protein bound with the drug (Supplementary Figure S4). Literature shows that fluorescence quenching takes place only when the ligand is located at a distance no greater than 10 nm from the tyrosyl and/or tryptophanyl residue in the protein. In this case, an exchange of energy takes place between the ligand and the fluorophore. The observed phenomenon indicates the participation of the tyrosyl group residues located in subdomains IIIA, IIA, IB, and/or IIB in forming the IBU-HSA complex, which is confirmed by Ghuman et al. in their work concerning specific places of drug binding with human serum albumin [19]. The data that were obtained allowed for determining the percent intensification (+)/quenching (−) of the IBU-HSA system fluorescence. The data are presented in Table 1.

Based on the data presented in Table 1 concerning the percent intensification (+) and quenching (−) of the tryptophanyl and tyrosyl residues (λex = 275 nm), tryptophanyl residue (λex = 295 nm) and tyrosyl residues (275–295 nm differential spectrum) it was observed that ibuprofen intensified HSA fluorophore fluorescence when the IBU-HSA system was excited by radiation of both wavelengths λex = 275 nm and λex = 295 nm. The fluorescence intensification percentage increased with the increased measurement temperature for all analyzed cases. However, a larger intensification was found when exciting the protein with a wavelength of λex = 295 nm. This means that the tryptophanyl residue is mainly responsible for the HSA fluorescence intensification. On the other hand, when exciting the IBU-HSA system with a wavelength of λex = 275 nm, exciting both tryptophanyl and tyrosyl residues, it was found that fluorescence intensification is much weaker than for a wavelength of λex = 295 nm. In order to verify the effect of the tyrosyl residue on the HSA quenching/intensification percentage, a differential spectrum for the IBU-HSA system was performed. The highest quenching value was found at a temperature of 310 K, while the lowest value was found at 308 K. This testifies to the participation of only tyrosyl residues in the formation of the IBU-HSA complex.

**Figure 4:**
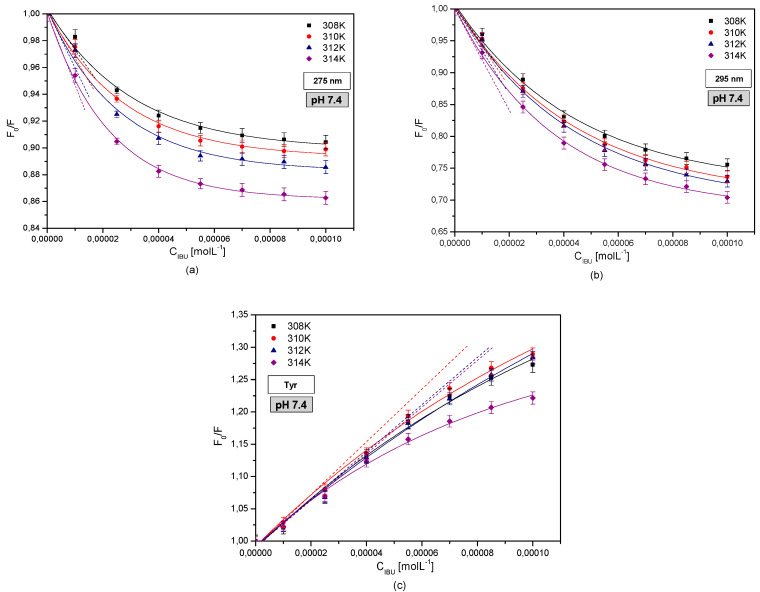
The Stern-Volmer plots of F_0_/F vs. C_IBU_ (M^−1^) for IBU-HSA complex in 308 K (■); 310 K (●); 312 K (▲); 314 K (◆), (**a**) λex = 275 nm—tryptophan + tyrosine; (**b**) λex = 295 nm—tryptophan; (**c**) differential spectrum—tyrosine, pH = 7.4.

Figure 4 shows original Stern-Volmer curves for the IBU-HSA complex excited by wavelengths of λex = 275 nm (Figure 4a) and λex = 295 nm (Figure 4b) and temperature ranges of 308–314 K, as well as original Stern-Volmer curves of the differential spectrum obtained for the system where only the albumin tyrosyl residues were excited (Figure 4c).

The original Stern-Volmer curves yield significant information concerning the dynamics and type of quenching/intensification of albumin fluorescence. The linear relationship F_0_/F = f([drug]) informs us about the dynamic quenching of the protein fluorophores within the specific subdomain that is responsible for the creation of the drug-albumin complex. A positive or negative deviation from the straight line indicates an additional static quenching effect [35,36].

The obtained Stern-Volmer curves indicate a positive deviation with respect to a straight line for excitations of the protein tryptophanyl residues in the entire analyzed ligand concentration range. For excitation of only the tyrosyl residues of HSA, a negative deviation with respect to the straight line was observed in a concentration range of 2.5 × 10^−5^–1.0 × 10^−4^ M^−1^ in the entire studied temperature range. This testifies to the fact that in the case of the IBU-HSA complex, a dynamic and static fluorophore fluorescence intensification effect takes place when exciting the tryptophanyl residues, yet in the case of exciting the tyrosyl residue, a dynamic quenching effect initially takes place, and above IBU concentrations of IBU 2.5 × 10^−5^ M^−1^ in the IBU-HSA complex, an additional static fluorescence quenching effect is observed.

The modified Stern-Volmer curves describe the interaction of ibuprofen with human serum albumin fluorophores, where the K_SV_ constant allowed for determining the availability of the fluorophores for the ligand, characterizing the distance between the ligand and the excited protein fluorophore. With increasing values of the Stern-Volmer constant K_SV_ the probability of ligand-protein complex creation via the nearing of the ligand to albumin increases. It must be remembered that the stronger the complex the weaker the therapeutic effect [35].

Based on a comparison of the linear regression equations, Stern-Volmer constant K_SV_ values were determined. The data are presented in Table 2.

Based on the presented Stern-Volmer constant values (Table 2), it may be stated that when exciting the IBU-HSA system with radiation with wavelengths of λex = 275 and λex = 295 nm (Supplementary Figure S5), the strongest complex is created at a temperature of 314 K while the weakest interaction between ibuprofen and human serum albumin was observed at a temperature of 308 K. When exciting only the tryptophanyl residue (λex = 295 nm), the Stern-Volmer constants are comparable in temperatures 310 and 312 K, which testifies to the same affinity to albumin. However, when exciting only the tyrosyl remainders, the highest Stern-Volmer constant value was found for a temperature of 314 K and the lowest at 308 K. From the obtained Stern-Volmer constant K_SV_ values it may be clearly stated that with increasing temperatures the affinity of tyrosyl and tryptophanyl residues for albumin increases. When exciting both tyrosyl and tryptophanyl HSA residues, the highest ibuprofen affinity for HSA, and therefore the lowest therapeutic effect, was found at a temperature of 314 K. The strongest therapeutic effect is exhibited by ibuprofen bound with albumin at a temperature of 308 K.

The study of the influence of temperature on metabolism and drug binding on the example of midazolam conducted by Miyamoto et al. confirmed its special importance. The results obtained in induced hypothermia at 28 °C (301 K) proved to be important. This emphasizes the particular importance of conducting research under non-physiological conditions [22].

In the study concerning the interaction between coenzyme Q_10_ and human serum albumin conducted by Peng et al. a similar conclusion to our study was observed [37]. It stated that the K_SV_ value increases with temperature. They also suggest that the fluorescence quenching might be dynamic. However, the presence of changes between the albumin absorption spectrum and the differential absorption spectrum between the HSA-CoQ_10_ complex and CoQ_10_, which was also observed, confirm the presence of static fluorescence quenching.

Based on data collected from emissive fluorescent spectra, Scatchard plots (Supplementary Figure S6), Klotz plots (Supplementary Figure S7), and Hill plots (Supplementary Figure S8) were created in order to determine the K_a_ association constants and the number of binding sites in the IBU-HSA system.

Scatchard plots provide detailed information about the association constant, the average number of drug molecules per one protein molecule, and the number of binding classes of IBU in subdomains IIA, IB, IIB, and/or IIIA [38].

Based on the obtained Scatchard plot (Supplementary Figure S6), it may be clearly stated that when exciting the IBU-HSA system with radiation of wavelengths of λex = 275 nm and λex = 295 nm, the curves are nonlinear. It is believed that in the surroundings of the tryptophanyl and tyrosyl residues many classes of binding sites exist, or the ligand-protein bond is non-specific [39]. A similar conclusion was made by Salem et al. in the study concerning the interaction between safranal and crocin with human serum albumin [40]. The nonlinear Scatchard plot proves that safranal and crocin bind to two types of binding sites on HSA. It states that they exhibit negative cooperativity—binding on one site decreases the affinity for binding on the other sites [41].

Based on the Klotz plots, we obtain information about the association (binding) constant values K_a_ and the number of binding sites, assuming only one class of binding site for binding the ligand to the protein. In the case of a larger number of binding site classes, the Klotz plots give information only about the average association constant values for these sites [42].

Based on the obtained Klotz plots (Supplementary Figure S7), it may be clearly stated that the relationship of 1/r vs. 1/[L_f_] shows a linear character when the IBU-HSA complex is excited by radiation of a wavelength of λex = 275 nm and λex = 295 nm in the entire temperature range. This testifies to the existence of one class of IBU binding sites in the albumin molecule.

In order to analyze protein-ligand interactions, the association constant K_a_ and the Hill coefficient were determined based on the Hill curve equation expressed by the relationship between log (𝑟/(1 − 𝑟)) and log [L_f_] (Supplementary Figure S8).

The analysis of the relationship between log (𝑟/(1 − 𝑟)) and log [L_f_] has shown that the Hill curves for the IBU-HSA complex at temperatures of 310 and 312 K with the tyrosyl and tryptophanyl residues being excited were similar. This testifies to a similar degree of cooperativity between the protein and the studied drug. A similar behavior was observed when exciting only the tryptophanyl residue in temperatures of 308 and 314 K as well as 310 and 312 K. However, when exciting only the tyrosyl residues, the Hill curves in the studied temperature range differ, which testifies to different degrees of cooperativity of HSA with IBU [43].

Based on the equation of the Hill curves that were obtained, the Hill coefficient values were determined, which are shown in Table 3.

By analyzing the association constant values K_a_ (Table 3) that were obtained, it was concluded that when exciting the IBU-HSA system with radiation with a wavelength of λex = 275 nm, the highest K_a_ value is found for the system at a temperature of 314 K. The lowest K_a_ is shown by the IBU-HSA system at a temperature of 308 K. Based on the analysis that was performed when both tyrosyl and tryptophanyl residues are being excited, it is believed that with an increase of temperature the ibuprofen-albumin binding association constant value increases. When exciting the IBU-HSA complex with a wavelength of λex = 295 nm it was observed that the highest association constant value as determined by the Klotz method is found for a temperature of 314 K and the lowest for a temperature of 310 and 312 K. When exciting only the human serum albumin tyrosyl residue, the highest association constant K_a_ value was observed for a temperature of 314 K and the lowest for 312 K. The analysis of association constants K_a_ with the use of the Hill method, exciting only the protein tryptophanyl residues (λex = 295 nm), showed a similar relationship between K_a_ and temperature. Exciting only the tyrosyl residues of albumin, the highest association constant was found for 314 K, similar as for the Klotz method. The lowest K_a_ value was found for a temperature of 308 and 312 K.

In order to determine the number of ligand molecules per one albumin molecule, the number of binding sites was determined using the Klotz method (Table 3). It was found that most ibuprofen molecules bind to one HSA molecule at a temperature of 314 K when exciting the tyrosyl and tryptophanyl residues and at 308 K when exciting only the tryptophanyl residues. It is believed that at temperatures of 308 K (λex = 275 nm) and 314 K (λex = 295 nm) IBU exhibits a greater affinity to the albumin fluorophores than at the other analyzed temperatures.

By analyzing the protein-ligand interaction with the use of Hill curves, Hill constants (*n_H_*) values were determined. For *n_H_* = 1 the binding of the ligand to the protein molecule is noncooperative. For *n_H_* > 1 there is a positive cooperative relationship. Binding the ligand in one place increases the affinity of the ligand to the rest of macromolecule binding sites. For *n_H_* < 1 means a reduction in the affinity of the ligands to the next binding site [44]. When exciting the IBU-HSA system with radiation with a wavelength of λex = 275 nm, no effect of temperature on the *n_H_* value was found. In the studied temperature range the results that were obtained were repeatable, and were in the range of 0.80 ± 0.12 ÷ 0.84 ± 0.08. When exciting the tryptophanyl residue of human serum albumin, the highest Hill constant values were found for temperatures of 310 and 314 K. By analyzing only the protein tyrosyl residue it was observed that the *n_H_* values are comparable in the studied temperature ranges and are in the range of 1.03 ± 0.05–1.08 ± 0.07. Based on the results that were obtained, it may be concluded that in the case of the IBU-HSA complex there is a negative coherent bond between the protein tryptophanyl residues and ibuprofen, which testifies to a weakening of the bond in the second-class binding site. On the other hand, when exciting only the tyrosyl residues of HSA, a positive cooperative relationship takes place: The binding of the ligand in one site increases its affinity to other protein binding sites [43].

### 2.2. The Effect of Human Serum Albumin pH on Binding with Ibuprofen

**Figure 5:**
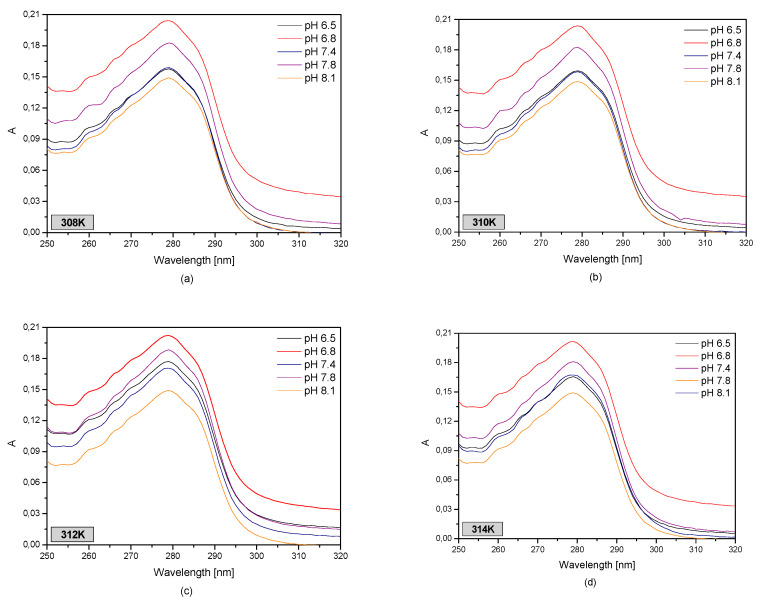
The spectrophotometric spectra of human serum albumin (5 × 10^−6^ M) in (**a**) 308 K; (**b**) 310 K; (**c**) 312 K; (**d**) 314 K for pH 6.5 (**‒**); pH 6.8 (**‒**); pH 7.4 (**‒**); pH 7.8 (**‒**); pH 8.1 (**‒**).

Using the spectrophotometric analysis in order to describe the structural changes in blood serum albumin caused by IBU (1 × 10^−5^–1 × 10^−4^ M) as a function of protein pH (pH 6.5–8.1) and temperature (T = 308–314 K), UV-VIS curves were created (Figure 5).

Based on Figure 5, it was concluded that the lowest absorbance value in the studied temperature range was found for an albumin solution with a pH of 8.1, while the highest was found for a pH of 6.8. Furthermore, it may be observed that the shape of the absorption curves of protein solution at pH of 7.4 and 6.5 is the same for temperatures of 308, 310, and 314 K. By analyzing the second order derivative of the albumin spectrum (data not shown), it was found that the pH value of the protein solution has an effect on changes in the HSA tertiary structure. In the studied temperature range it was observed that for wavelengths of 250–270 nm, which is the range encompassing the phenylalanine residues, only in the case of the solution with a pH of 7.8 changes occurred in the protein structure. In the wavelength ranges of 293–305 nm and 270–293 nm, which describe the tryptophanyl and tyrosyl resides, it was found that the curves for pH values of 6.5 and 6.8 are the same. In the remaining cases structural changes in albumin took place.

**Figure 6:**
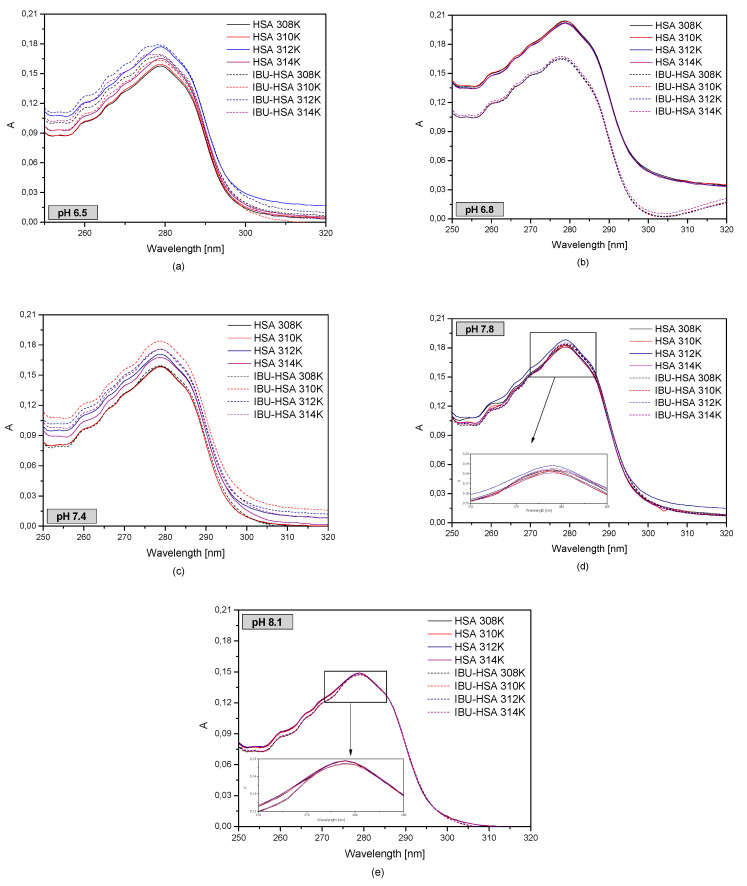
The spectrophotometric spectra of human serum albumin (5 × 10^−6^ M) at various concentrations of ibuprofen (1 × 10^−5^–1 × 10^−4^ M) in temperature T = 308 K (**‒**); T = 310 K (**‒**); T = 312 K (**‒**); T = 314 K (**‒**) for (**a**) pH 6.5; (**b**) pH 6.8; (**c**) pH 7.4; (**d**) pH 7.8; (**e**) pH 8.1.

Figure 6 shows the first order derivative of the human serum albumin spectrum with and without ibuprofen in different albumin temperatures and pH values.

When analyzing the first (Figure 6) and second order derivatives (Supplementary Figure S9) with and without ibuprofen, it was concluded that changes in the temperature influence the HSA absorbance in the studied albumin pH range. By analyzing the albumin solution with a pH of 6.5 it was concluded that with increasing temperature, the changes in absorbance in the presence of IBU decrease. In the case of an HSA solution with a pH of 6.8, significant changes in absorbance were observed after the addition of ibuprofen, while for the solution with a pH of 7.4 it was found that at a temperature of 308 K the addition of IBU had no effect on HSA absorbance. The largest changes were observed at a temperature of 310 K. When analyzing an HSA solution with a pH of 7.8, it was observed that the addition of IBU causes a decrease in the HSA absorbance value. In the other temperatures no significant changes were observed.

**Figure 7:**
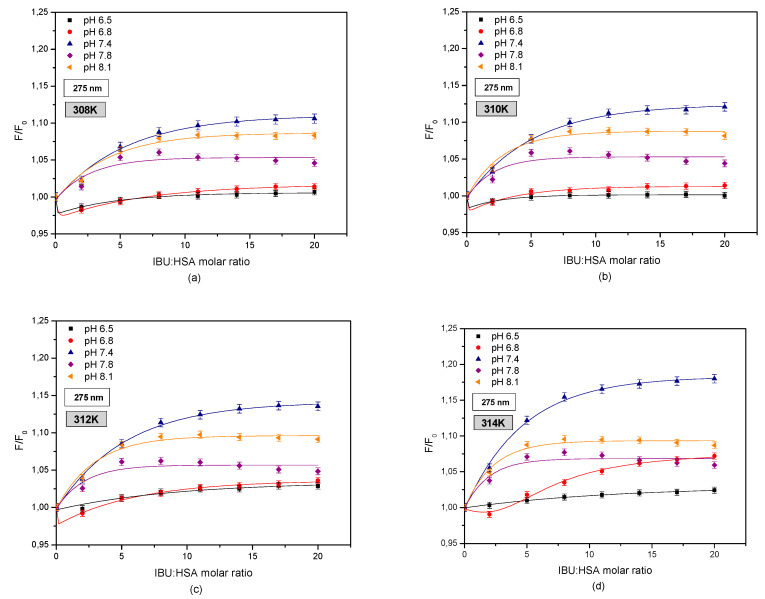
Fluorescence intensification/quenching curves of human serum albumin (5 × 10^−6^ M) at various concentrations of ibuprofen (1 × 10^−5^–1 × 10^−4^ M) in (**a**) 308 K; (**b**) 310 K; (**c**) 312 K; (**d**) 314 K for pH 6.5 (■); pH 6.8 (●); pH 7.4 (▲); pH 7.8 (◆); pH 8.1 (◄), λex = 275 nm.

Comparing the second derivatives of the absorbance spectrum (Supplementary Figure S9), it was observed that in the wavelength ranges of 270–293 nm and 250–270 nm in the case of albumin solutions with a pH of 6.5 and 6.8, there were differences between the HSA spectrum and the IBU-HSA complex spectrum. When analyzing the UV-VIS spectrum of a pH 7.4 albumin solution, subtle differences were observed in the curves of HSA and IBU-HSA only in the wavelength range of 270–293 nm. At higher pH values no differences were found. In order to analyze the effect of HSA and pH on binding with ibuprofen, fluorescence intensification/quenching curves were created in the temperature range of 308–314 K when exciting the IBU-HSA complex with radiation with a wavelength of λex = 275 nm (Figure 7).

When analyzing the curves showing the relationship of HSA fluorescence $\left(F/F_{0}\right)$ vs. the IBU-HSA molar ratio (Figure 5), the largest increase in fluorophore fluorescence intensity in the entire concentration and temperature range was found for albumin with a pH of 7.4. In the case of HSA solutions with pH values of 8.1 and 7.8, the increase in fluorophore fluorescence is lower than that for the solution with a pH of 7.4. The lowest $\left(F/F_{0}\right)$ values were observed for albumin solutions with pH values of 6.5 and 6.8. Furthermore, when exciting both the tryptophanyl and tyrosyl residues in HSA at pH values of 6.5 and 6.8, it was found that at a IBU-HSA molar ratio of 2:1 a decrease in the fluorophore fluorescence took place as a result of an energy exchange between IBU and the tryptophanyl and/or tyrosyl residues being located no more than 10 nm apart from the ligand. This testifies to the participation of tyrosyl residues located in subdomains IIIA, IIA, IB, and/or IIB in creating the IBU-HSA complex [19]. The increase in the IBU concentration contributes to an increase in the HSA fluorophore fluorescence intensity.

**Figure 8:**
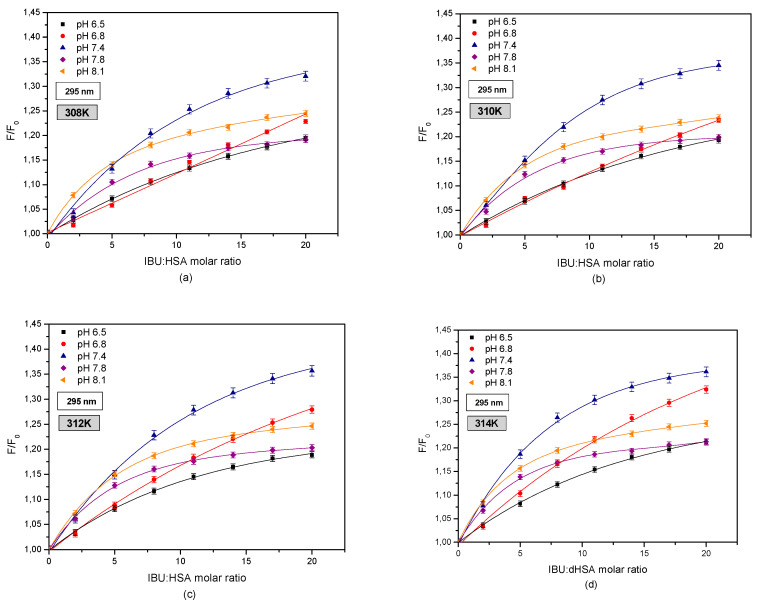
Fluorescence intensification/quenching curves of human serum albumin (5 × 10^−6^ M) at various concentrations of ibuprofen (1 × 10^−5^–1 × 10^−4^ M) in (**a**) 308 K; (**b**) 310 K; (**c**) 312 K; (**d**) 314 K for pH 6.5 (■); pH 6.8 (●); pH 7.4 (▲); pH 7.8 (◆); pH 8.1 (◄), λex = 295 nm.

In order to evaluate the effect of tryptophanyl residue on the creation of the IBU-HSA complex, curves illustrating the relationship between HSA fluorescence $\left(F/F_{0}\right)$ and the IBU-HSA molar fraction were created for exciting the IBU-HSA system with radiation with a wavelength of λex = 295 nm in the temperature range of 308–314 K (Figure 8).

By comparing these curves as shown in Figure 5 and Figure 6, a difference was observed between the intensity of exciting human serum albumin fluorescence. At this stage it may be surmised that both tyrosyl and tryptophanyl residues play a role in creating the IBU-HSA complex in the entire studied albumin solution pH range. The strongest excitation of fluorophore fluorescence in the analyzed temperature range was found for the HSA solution with a pH of 7.4, excited by radiation with a wavelength of λex = 295 nm. Below an IBU-HSA molar ratio of 5:1 (T = 308 and 310 K) and 2:1 (T = 312 and 314 K) it was found that the $\left(F/F_{0}\right)$ value is higher for an HSA solution with a pH of 8.1 than for a solution with a pH of 7.4.

Furthermore, when analyzing the fluorescence curves shown in Figure 6 it was concluded that above an IBU-HSA molar ratio of 20:1, the fluorescence value for HSA solutions with a pH of 6.5 and 7.8 are comparable.

**Figure 9:**
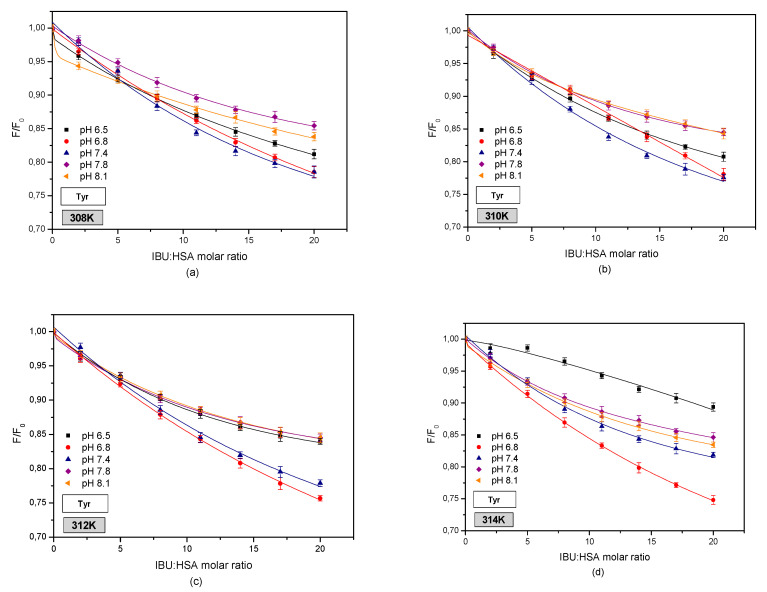
Fluorescence intensification/quenching curves of tyrosine in human serum albumin (5 × 10^−6^ M) at various concentrations of ibuprofen (1 × 10^−5^–1 × 10^−4^ M) in (**a**) 308 K; (**b**) 310 K; (**c**) 312 K; (**d**) 314 K for pH 6.5 (■); pH 6.8 (●); pH 7.4 (▲); pH 7.8 (◆); pH 8.1 (◄).

By analyzing the role tyrosyl (Tyr) residues play in creating the IBU-HSA bond, differential spectra were created for the protein-drug system (Figure 9).

Based on fluorescence quenching curves for ibuprofen influence on HSA tyrosyl residues, it was concluded that at a temperature of 308 K and below an IBU-HSA molar ratio of 5:1 ibuprofen most effectively quenches tyrosyl residue fluorescence at a pH equal to 8.1. The increase in ibuprofen concentration causes an increase in the albumin tyrosyl residue quenching at a pH of 7.8.

When exciting the tyrosyl residues at a temperature of 310 K, it was observed that the strongest quenching effect in the entire studied IBU concentration range was found for the albumin solution with a pH of 7.4, whereas the weakest quenching effect was found in the case of HSA with pH values of 7.8 and 8.1. The fluorescence quenching curves for albumin tyrosyl residues for pH values of 7.8 and 8.1 have a similar course.

At temperatures of 312 and 314 K, ibuprofen most effectively quenches HSA fluorophore fluorescence at a pH of 6.8, and the weakest quenching is found for an albumin solution with a pH of 6.5 for a temperature of 314 K and for solutions with pH values of 7.8 and 8.1 for a temperature of 312 K.

By plotting the albumin fluorescence differential spectra shown in Figure 9, it can be clearly concluded that mainly tyrosyl residues play a role in creating the IBU-HSA complex [19].

Based on the curves of HSA fluorescence as a function of the IBU-HSA molar ratio, fluorescence percentage intensification (+)/quenching (−) values were determined. The data are shown in Table 4.

By analyzing these values, it was observed that a pH 7.4 albumin solution most effectively intensified tryptophanyl residue fluorescence in the studied temperature range in the case of exciting the IBU-HSA system with radiation with a wavelength of λex = 295 nm. Furthermore, it was concluded that only in the case of albumin solutions with pH values of 6.8, 7.4, and 7.8, the percent intensification of HSA tryptophanyl residue fluorescence increased with temperature.

When exciting the IBU-HSA system with radiation with a wavelength of λex = 275 nm, exciting both the tyrosyl and tryptophanyl residues, it was observed that only in the case of HSA solutions with pH values of 6.8 and 7.4, the percentage fluorophore fluorescence intensification increased with temperature.

When analyzing the percentage fluorescence values of human serum albumin tyrosyl residues in the IBU-HSA complex obtained via differential spectra, it was found that regardless of the solution pH value and temperature, HSA fluorophore fluorescence quenching takes place. In the case of HSA solution with pH values of 6.5, 7.4, and 7.8, the highest fluorophore fluorescence quenching values were observed at a temperature of 310 K, while for HSA solutions with pH values of 6.8 and 8.1, the highest value was found for a temperature of 314 K.

**Figure 10:**
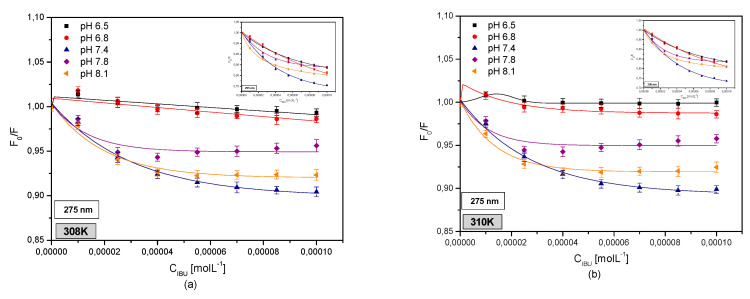

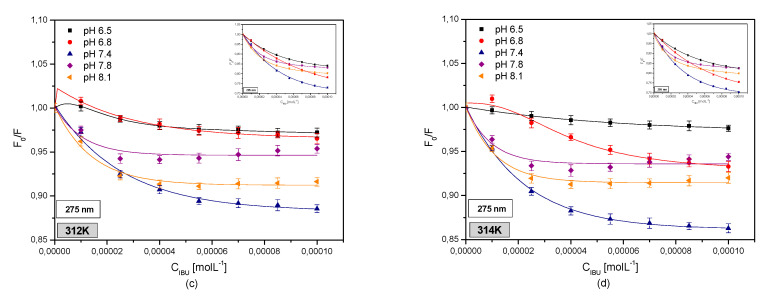
The Stern-Volmer plots of F_0_/F vs. C_IBU_ (M^−1^) for the IBU-HSA complex in (**a**) 308 K; (**b**) 310 K; (**c**) 312 K; (**d**) 314 K for pH 6.5 (■); pH 6.8 (●); pH 7.4 (▲); pH 7.8 (◆); pH 8.1 (◄), λex = 275 nm and λex = 295 nm (in insert).

Figure 10 shows original Stern-Volmer curves for the IBU-HSA system excited by radiation with a wavelength of λex = 275 nm and λex = 295 nm in the temperature range of 308–314 K for protein solutions in the pH range of 6.5–8.1.

Based on the Stern-Volmer curves (Figure 10) it was observed that when exciting the IBU-HSA systems at pH values of 7.4, 7.8, and 8.1 with λex = 275 nm wavelength radiation, a dynamic fluorophore fluorescence quenching takes place below an IBU concentration of C_IBU_ = 2.5 × 10^−5^ M. A further increase in the IBU concentration contributes to a further static fluorescence quenching effect. In the case of an albumin solution with a pH of 6.5 in the entire studied temperature range and an albumin solution with a pH of 6.8 at temperatures of 308, 310, and 312 K, a static and dynamic fluorophore fluorescence quenching effect takes place after binding with ibuprofen. On the other hand, at a temperature of 314 K the IBU-HSA system with a pH of 6.5 shows only a dynamic fluorophore fluorescence quenching effect. When exciting the IBU-HSA complex by radiation with a wavelength of λex = 295 nm, a linear relation $F/F_{0}=f\left[\mathrm{drug}\right]$ was observed for an albumin solution with a pH of 6.5 below a concentration of C_IBU_ = 4.0 × 10^−5^ M, a pH of 6.8 below C_IBU_ = 5.5 × 10^−5^ M (T = 308, 310, and 312 K) and C_IBU_ = 4.0 × 10^−5^ M (T = 314 K), a pH of 7.4 below C_IBU_ = 4.0 × 10^−5^ M, and pH values of 7.8 and 8.1 below C_IBU_ = 2.5 × 10^−5^ M in the entire studied temperature range. This testifies to a dynamic intensification of HSA fluorophore fluorescence. An increase in the ibuprofen concentration contributes to the presence of an additional static fluorescence intensification effect [36].

By analyzing the Stern-Volmer curves for HSA tyrosyl residues (data not shown) it was found that in the case of HSA solutions with a pH of 6.5 below an IBU concentration of C_IBU_ = 4.0 × 10^−5^ M at temperature of 308 and 310 K and C_IBU_ = 2.5 × 10^−5^ M at a temperature of 312 K a linear relationship $F/F_{0}=f\left[\mathrm{drug}\right]$ was observed, which testifies to a dynamic protein fluorophore fluorescence quenching. A similar effect was observed for albumin solutions with a pH of 7.4 below C_IBU_ = 7.0 × 10^−5^ M (T = 308, 310, and 312 K) and C_IBU_ = 4.0 × 10^−5^ M (T = 314 K); pH 7.8 below C_IBU_ = 7.0 × 10^−5^ M (T = 308 K) and C_IBU_ = 4.0 × 10^−5^ M (T = 310–314 K); and for pH 8.1 below C_IBU_ = 4.0 × 10^−5^ M (T = 310–314 K). An increase in the ibuprofen concentration contributes to the presence of additional static fluorophore fluorescence quenching of the protein tyrosyl residues (a negative deviation from a straight line). In the case of a pH 6.8 albumin solution, a linear $F/F_{0}=f\left[\mathrm{drug}\right]$ was found in the entire studied IBU concentration and temperature range. However, in the case of a protein solution with a pH of 6.5 at a temperature of 314 K and for a pH 8.1 solution at a temperature of 308 K, a negative deviation from the straight line $F/F_{0}=f\left[\mathrm{drug}\right]$ in the entire studied IBU concentration range took place, which testifies to a dynamic and static quenching of albumin fluorophore fluorescence.

Based on the Stern-Volmer curves, modified Stern-Volmer curves were created, from which Stern-Volmer constants K_SV_ were determined. The data are presented in Table 5.

Based on these constants K_SV_ (Table 5), it was found that when exciting the IBU-HSA system with a wavelength of λex = 275 nm, the K_SV_ value increased with temperature for albumin solution pH values of 6.5, 7.4, 7.8, and 8.1. It was also observed that with the increasing pH values the Stern-Volmer constant value increased in the studied temperature range. When exciting the HSA tryptophanyl resides, similar conclusions were reached. Furthermore, it was found that when exciting the system with radiation with a wavelength of λex = 275 nm and λex = 295 nm, the lowest affinity of ibuprofen for albumin occurs at a pH of 6.8, while the highest affinity occurs at a pH of 8.1. The greater the affinity of the drug to the protein, the smaller the therapeutic effect [35]. A similar phenomenon was observed by Samira Ranjbar in his work concerning isoimperatorin-HSA interaction. He explained that the increase in the Stern-Volmer constant with increasing temperature indicates that the involved binding forces are mainly hydrophobic interactions (endothermic apolar interactions are strengthened with increasing temperature) [45].

In order to evaluate the role of tyrosyl residues in creating the IBU-HSA complex, Stern-Volmer constants were determined, based on which it was found that in the case of human serum albumin with pH values of 6.5 and 6.8 in the entire studied temperature range and for a pH of 8.1 in the temperature range of 308–312 K the K_SV_ value decreased with temperature. The decrease in the K_SV_ with increasing temperature indicates a lowering of the availability of ibuprofen for the human serum albumin tyrosyl residues. This phenomenon suggests structural changes in human serum albumin due to a temperature effect [46]. Analyzing HSA solutions with pH values of 7.4 and 7.8 (except T = 314 K) it was found that with increasing temperature the Stern-Volmer constant value increases, and therefore a stronger complex is created with a lower therapeutic effect [35].

In order to determine the K_a_ association constant and the number of binding sites in the IBU-HSA complex, Scatchard, Klotz, and Hill plots were created (data not shown). Based on the Scatchard plots, it was found that regardless of the pH and temperature of the protein, when exciting the IBU-HSA complex with a wavelength of λex = 275 nm and λex = 295 nm, the curve is non-linear, which may indicate the presence of many classes of binding sites in the region of the tryptophanyl and tyrosyl residues or that the character of ligand-protein bond is non-specific [39].

Based on the Klotz plots created for the IBU-HSA complex, and the differential spectra when exciting only the HSA tryptophanyl residues, the association constant (K_a_) and the number of binding sites (*n*) were determined (Table 6).

Based on the association constant K_a_, values that were obtained when exciting the tryptophanyl and tyrosyl residues (λex = 275 nm), it was observed that in the case of pH values of 6.5 (in the temperature range of 310–314 K), 6.8, and 8.1 the association constant value decreases with the increasing temperature. When subjecting the IBU-HSA system with pH values of 7.4 and 7.8 to a wavelength of λex = 275 nm, the K_a_ increases with increasing temperature. Furthermore, when analyzing the obtained association constant values, it was found that the highest K_a_ values occur in the case of HSA solutions with pH values of 7.8 and 8.1, with the lowest occurring for HSA with a pH of 7.4. Therefore, the higher the association constant value, the lower the therapeutic effect [35].

By subjecting the IBU-HSA system to a wavelength of λex = 295 nm, it was observed that when exciting the tryptophanyl residue of pH 6.5 and 7.8 albumin, the K_a_ value increases with temperature. On the other hand, in the case of pH 7.8 and 8.1 HSA solutions in the temperature range of 308–312 K the association constant value decreases with increasing temperature. When exciting the tryptophanyl residues of pH 7.4 albumin, the lowest K_a_ values were found for temperatures of 310 and 312 K. The highest values were found for temperatures of 308 and 314 K. Furthermore, it was observed that the lowest association constant in the studied temperature range was found for albumin solutions with a pH of 6.5 and 6.8 and the highest values were found for pH values of 7.8 and 8.1.

When analyzing the association constant values for human serum albumin tyrosyl residues, no relationship was found between temperature and the association constant in the studied temperature range for albumin pH values of 6.5, 6.8, and 7.4. Comparing the K_a_ values in the temperature range 308–312 K a decrease in the association constant with increasing temperature may be observed for albumin with a pH of 8.1. On the other hand, for an HSA solution with a pH of 7.8, the opposite tendency was found, i.e., the association constant increased with increasing temperature. Additionally, based on the results, it may be concluded that the lowest K_a_ values in the studied temperature and pH range may be observed in the IBU-HSA system where the pH is equal to 7.4.

Yassen and El-Ghossain conducted studies on the interaction of three drugs (diclofenac sodium, furosemide, and dexamethasone) with human serum albumin at 25 °C (298 K) and at different pH values 6.0–8.0. It was found that with increasing temperature the value of the association constant decreases. The association constant at each pH was calculated with using the modified Stern-Volmer equation. They suggested that probably the temperature of the analysis is of great importance for the value of the binding constant between the ligand and the blood plasma protein [47].

In order to determine the number of ibuprofen molecules *n* per one HSA molecule (Table 6), it was found that when stimulating the complex with wavelengths of λex = 275 nm, the greatest number of ibuprofen molecules per one HSA molecule occurs when the protein pH is equal to 6.8, and the smallest number is found for a solution with a pH of 6.5. In the case of albumin solutions with a pH of 7.4 at temperatures of 308–312 K and a pH of 8.1 at temperatures of 312–314 K the number of binding sites *n* is close to unity. This indicates the lack of a specific ibuprofen binding site in the HSA molecule. Similar values were obtained when exciting the IBU-HSA complex with λex = 295 nm wavelength radiation in the case of albumin solution pH values of 6.5, 6.8, and 8.1 in the entire studied temperature interval, for pH 7.4 in the temperature range of 310–314 K, and for pH 7.8 in the temperature range of 312–314 K. By analyzing the number of sites where ibuprofen binds with the tyrosyl residue, it may be observed that in the studied albumin pH and temperature range, one specific ligand binding site exists in the protein molecule.

In order to determine the degree of cooperativity of the protein with ibuprofen, Hill plots for the IBU-HSA complex were created (data not shown). Based on the Hill curve equation, the association constants K_a_ and the Hill coefficient were determined. The data are presented in Table 7.

When analyzing the association constant K_a_ obtained based on the IBU-HSA Hill curves, it was concluded that when exciting the IBU-HSA complex with λex = 275 nm wavelength radiation, the association constant value K_a_ increases with temperature in the case of HSA with a pH of 7.4 and 8.1. A similar phenomenon was found in the IBU-HSA complex where the albumin pH was 7.8 and at the temperature range of 308–312 K. On the other hand, in the case of an HSA solution with a pH of 6.8 and in the temperature range of 310–314 K it was found that with increasing temperature the K_a_ value decreases. When analyzing the association constant for an albumin solution pH of 6.5, it was found that the highest K_a_ value, and therefore the lowest therapeutic effect occurs at a temperature of 312 K ((6.01 ± 1.46) × 10^4^ M^−1^), while the lowest K_a_ value was found at a temperature of 314 K ((0.35 ± 0.08) × 10^4^ M^−1^).

When subjecting the IBU-HSA complex to radiation with a wavelength of λex = 295 nm, it was found that in the case of HSA with pH values of 6.5 and 7.8, the K_a_ increased with increasing temperature. The opposite relationship was observed when analyzing the IBU-HSA complex where the albumin pH values were 6.8 (in the entire studied temperature interval), 7.4 and 8.1 (in the temperature range of 308–312 K).

When comparing the association constant determined for albumin tyrosyl residues in the IBU-HSA complex, it was observed that in the case of an HSA solution with pH values of 6.5, 6.8 (in the temperature range of 310–314 K), and 7.6 (in the temperature range of 308–312 K), the K_a_ value increased with increasing temperature. On the other hand, when analyzing the albumin solution with a pH of 8.1, it was found that in the temperature range of 308–312 K the association constant decreases with increasing temperature. Analyzing the association constants K_a_ per the Hill method for the IBU-HSA system with an albumin pH of 7.4, it was observed that the highest K_a_ value occurs at a temperature of 314 K ((0.57 ± 0.03) × 10^4^ M^−1^), while the lowest value occurs at 308 K ((0.10 ± 0.02) × 10^4^ M^−1^) and 312 K ((0.07 ± 0.03) × 10^4^ M^−1^). Furthermore, comparing the obtained results with the association constant values of the remaining albumin solutions in the pH range of 6.5–8.1 it was found that ibuprofen exhibits the lowest affinity to the albumin tyrosyl remainders, and therefore the greatest therapeutic effect in the studied temperature range, in the case of albumin solutions with a pH of 7.4.

When analyzing the protein-ligand interaction with the use of the Hill method, the Hill coefficient (*n_H_*) was determined. When exciting the tyrosyl and tryptophanyl residues (λex = 275 nm), it was observed that in the case of pH 6.5 (in the temperature of 308 and 314 K), 7.4, 8.1 (in the entire studied temperature interval), the Hill coefficient was less than unity, which testifies to a negative cooperative bonding, which indicates a weakening of the bond in the second-class bonding site. When analyzing the protein solution with pH values of 6.8, 7.8, and 6.5 at a temperature of 310 K the *n_H_* value is greater than unity. This testifies to the binding of the ligand at one site which facilitates binding in the next site. Independent ligand-protein binding takes place at a temperature of 312 K, when the HSA pH is 6.5. This indicates that the ligand-protein bond strength is not dependent on other ligand molecules already bound with the protein.

When exciting the IBU-HSA system with λex = 295 nm wavelength, is was observed that in the case of HSA with pH values of 7.4, 7.8, and 8.1, a negative cooperative binding takes place, which indicates the weakening of ibuprofen bonding in the second-class HSA binding site. Independent ibuprofen binding takes place in the range of 308–314 K for albumin pH values equal to 6.5 and at temperatures of 312 and 314 K for a pH of 6.8. On the other hand, for pH 6.8 albumin at temperatures of 308 and 310 K, the Hill constant is larger than unity, which indicates a facilitation of ligand binding at the second site by binding at the first site.

By analyzing the HSA tyrosyl residues it was observed that in the case of the two extreme protein pH values, i.e., 6.5 (T = 314 K) and 8.1 (T = 308 K), positive coherent bonds are created, which testify to the binding of the ligand in one site facilitating the binding at the second site. In the other cases, the Hill interaction coefficient is close to unity, which indicates the presence of only one specific ibuprofen binding site in the albumin molecule [42].

## 3. Materials and Methods

### 3.1. Chemicals

Human serum albumin (HSA), fraction V were obtained from MP Biomedicals LLC (Illkirch, France). Ibuprofen (IBU), sodium dihydrogen phosphate (Na_2_HPO_4_), and dipotassium phosphate (K_2_HPO_4_) were purchased from Sigma-Aldrich Chemical Co. (Darmstadt, Germany). Tris(hydroxymethyl)aminomethane (TRIS), hydrochloric acid (HCl), and methanol were purchased from POCH S.A. (Gliwice, Poland). All reagents and solvents were of analytical reagent grade.

### 3.2. Solutions and Sample Preparation

A 0.05 M phosphate buffered saline (PBS) solution at pH 6.5, 6.8, 7.4, and 0.05 M tris(hydroxymethyl)aminomethane hydrochloride buffered (TRIS-HCl) solution at pH 7.8 and 8.1 to mimic physiological conditions was prepared by dissolving 1.4790 g of K_2_HPO_4_ and 1.9800 g of NaH_2_PO_4_ (pH 6.5), 2.1750 g of K_2_HPO_4_ and 1.5000 g of NaH_2_PO_4_ (pH 6.8), 3.4836 g of K_2_HPO_4_ and 0.6000 g of NaH_2_PO_4_ (pH 7.4), 87.0 mL 0.1 M HCl and 125.0 mL 0.2 M TRIS (pH 7.8), 125.0 mL 0.1 M HCl and 125.0 mL 0.2 M TRIS (pH 8.1) with the appropriate amount of purified water added to obtain 500 mL of total volume. For human serum albumin-drug interaction analysis a stock solution of ibuprofen in methanol (1.5 × 10^−2^ M) were prepared. A stock solution of human serum albumin (HSA) at the concentration of 5 × 10^−6^ M were prepared in PBS at pH 6.5, 6.8, 7.4 and TRIS-HCl at pH 7.8 and 8.1.

### 3.3. Fluorescence and UV-VIS Spectra

Fluorescence and absorption spectra were recorded in the temperature range between 308 and 314 K and pH albumin solution between 6.5 and 8.1 with 1 × 1 × 4 cm quartz cells. Absorption spectra were recorded on the JASCO V-760 spectrophotometer (Jasco International Co., Ltd., Tokyo, Japan) in the wavelength range between 250–400 nm. The fluorescence emission spectra were recorded on the JASCO fluorescence spectrophotometer FP-6500 (Jasco International Co., Ltd., Tokyo, Japan) equipped with a Peltier thermostat. The fluorescence emission spectra were collected in the wavelength range between 285–450 nm (λex = 275 nm) and 305–450 nm (λex = 295 nm). Widths of both the excitation and emission slit were set at 3.0 nm. Absorption second derivative spectra were obtained by the use of the Spectra Analysis program (Spectra Manager, version 1.55.00, Jasco International Co., Ltd., Tokyo, Japan) using Savitzky and Golay algorithm, second order of polynomial, and 15 data points. To obtain the complex IBU-HSA, the human serum albumin solution (5 × 10^−6^ M^−1^) was titrated directly into the cuvette by the addition of increasing aliquots of IBU stock solution (1.5 × 10^−2^ M^−1^). Light scattering caused by buffer was subtracted from the samples fluorescence using software JASCO (Spectra Manager). The intensity of fluorescence was corrected for the inner filter effect [32].

Based on fluorescence data, the fluorescence curves of human serum albumin (HSA) in the presence of ibuprofen have been plotted. The intensification/quenching effect of HSA fluorescence was analyzed on the basis of the Stern-Volmer equation. The Stern-Volmer plot describes dynamic and/or static movements of ligand in protein fluorescence quenching. The Stern-Volmer equation describes the relationship between ligand concentration and quenching/intensification fluorescence of albumin fluorophores [46] (Equation (1)):

(1) $$\frac{F_{0}}{F}=1+K_{\mathrm{SV}}\cdot \left[L\right]$$

where F_0_ and F are the fluorescence intensities of IBU-HSA in the absence and presence of ligand; K_SV_ is the Stern-Volmer constant [M^−1^]; and [L] is the concentration of ligand (IBU) [M^−1^].

To analyze the interactions between ibuprofen and human serum albumin, the Stern-Volmer (K_SV_) and association (K_a_) constants have been determined. The Stern-Volmer constant K_SV_ was calculated using the Stern-Volmer equation modified by Lehrer [48] (Equation (2)):

(2) $$\frac{F_{0}}{\Delta F}=\frac{1}{\left[L\right]}\cdot \frac{1}{f_{a}}\cdot \frac{1}{K_{\mathrm{SV}}}+\frac{1}{f_{a}}$$

where ΔF is the difference between the fluorescence intensities of HSA in the absence (F_0_) and presence (F) of ligand; and *f_a_* is the fractional maximum fluorescence accessible for the quencher.

Association constants K_a_ were calculated by the use of Scatchard (Equation (3)) and Klotz curves (Equation (4)):

(3) $$\frac{r}{\left[L_{f}\right]}=n\cdot K_{a}-K_{a}\cdot r$$

(4) $$\frac{1}{r}=\frac{1}{n}+\frac{1}{n\cdot K_{a}\cdot \left[L_{f}\right]}$$

where *n* is the number of binding sites in the albumin molecule; [L*_f_*] is the free drug concentration; and r is the number of ligands bound to one protein molecule.

The Scatchard curve (Equation (3)) describes the interaction between the protein molecule and ligand. It is used to determine the number of binding sites for the independent class of drug binding sites in the albumin molecule [38,49].

Klotz curves (Equation (4)) allows the determination of association constants K_a_ and the number of drug molecules bound to one protein molecule (*n*) [42,50].

Based on the Hill equation association constants K_a_ and interaction coefficients $n_{H}$ were determined (Equation (5)) [44]:

(5) $$log\left(\frac{r}{1-r}\right)=n_{H}\cdot log\left[L_{f}\right]+log\;K_{a}$$

where *n_H_* is the Hill’s coefficient.

### 3.4. Statistical Analysis

In this work, all experiments and measurements were carried out in triplicate, and data were expressed as a mean ± standard deviation (SD). The resulting data were analyzed by the use of OriginPro 8.5.0 SR1 (OriginLab Corporation, Northampton, MA, USA).

## 4. Conclusions

Human serum albumin (HSA) is the most common blood protein. Pathological states taking place in the human body, such as abnormal blood serum pH or temperature, significantly influence the interaction of the drugs with human serum albumin, which was confirmed by studies performed with the use of fluorescent spectroscopy and UV-VIS spectrophotometry. For performing an analysis of the IBU-HSA interaction at the different blood pH value of 6.5, 6.8, 7.4, 7.8, and 8.1 and temperatures 308, 310, 312, and 314 K, UV-VIS spectrophotometry was utilized. Based on this analysis, it was found that the pH and temperatures of human serum albumin affected the UV-VIS curves. There is change in the protein absorbance (Figure 5 and Figure 6). When analyzing the second derivative of the absorption, the effect of HSA pH on structural change in the area of albumin tryptophanyl and/or tyrosyl groups was observed at a temperature range of 308–314 K (Supplementary Figures S2 and S9).

By utilizing spectrophotometric analysis of the protein-ligand interaction, an increase in the hydrophobic character of the albumin tryptophanyl and/or tyrosyl group surrounding was found in the area of subdomains IIA, IIIA, IB, and/or IIB (Supplementary Figure S3). Comparing the protein fluorescence curves at pH values of pH 7.4, 7.8, and 8.1 shows that by interacting with the protein molecule, ibuprofen breaks the Trp-Trp hydrogen bonds, which causes an increase in the number of free Trp molecules in the system. The number of free Trp molecules in the IBU-HSA system increases with temperature (Table 4). This causes an increase in the tryptophanyl residue fluorescence intensity. The highest increase was found in the case of an HSA solution with a pH of 7.4. The shift in the fluorescence band of tryptophanyl residue testifies to tryptophan-ibuprofen interactions with a free tryptophan molecule (Figure 3 and Figure 7). When analyzing the fluorescence differential curves at the studied protein pH, it was found that mainly the tyrosyl residues participate in creating the IBU-HSA complex (Supplementary Figure S4 and Figure 9). Based on the analysis of Stern-Volmer curves it was found that fluorescence intensification is static and dynamic in the studied concentration and temperature range. When exciting only the tyrosyl residues of pH 6.5, 7.4, 7.8, and 8.1 albumin, initially only dynamic fluorophore fluorescence quenching was observed, and then an additional static HSA quenching effect. In the case of pH 6.8 solution, only a dynamic HSA fluorophore fluorescence quenching takes place (Figure 4 and Figure 10).

By utilizing modified Stern-Volmer curves, Stern-Volmer constants were determined, which describe the affinity of the ligand to a protein. Based on them, it was found that in the studied pH range HSA the tyrosyl and tryptophanyl residue K_SV_ value increases with increasing temperature, forming a stronger complex (Supplementary Figure S5 and Table 5). Therefore, ibuprofen is released from the IBU-HSA complex more slowly, exhibiting a longer but weaker effect. In the case of exciting only HSA tyrosyl residues at pH values of 6.5, 6.8, and 8.1, it was observed that the K_SV_ decreases with increasing temperature.

In order to deepen the analysis of the effect of temperature and pH on the protein-ligand bond strength, Klotz and Hill plots were created. Based on them it was concluded that with increasing temperature, the association constant of the IBU-HSA (pH 7.4, 7.8) increases with increasing temperature when excited by a wavelength of λex = 275 nm, which indicates an increase in the stability of the complex. Analyzing the HSA solution with pH values of 6.5, 6.8, and 8.1, it was observed that with increasing temperature the association constant decreases, strengthening the therapeutic effect. The association constant of pH 7.4 albumin is the lowest in the studied serum temperature and pH range, which indicates the best therapeutic effect. Similar conclusions were formed when analyzing the K_a_ values only for the protein tyrosyl residues.

When analyzing the number of binding sites, it was observed that when exciting the IBU-HSA system with λex = 275 nm wavelength radiation, the highest number of ibuprofen molecules per one albumin molecule occurs for a pH of 6.8, with the lowest number occurring for a pH of 6.5. When analyzing the tyrosyl and tryptophanyl residues, the presence of one specific ibuprofen binding site was found in human serum albumin.

When creating the Hill plots for the albumin tyrosyl residues, it was found that regardless of the protein pH, the Hill coefficient *n_H_* is close to unity, which indicates the presence of one specific ibuprofen binding site in human serum albumin. Similar conclusions were formed when exciting the tryptophanyl residues at pH values of 6.5 and 8.1 (Table 7).

## Figures and Tables

**Table 1:** Percent intensification (+)/quenching (−) of the IBU-HSA system fluorescence.

IBU-HSA	λex = 275 nm Trp + Tyr	λex = 295 nm Trp	Tyr
308 K	+10.57%	+32.36%	−21.79%
310 K	+11.24%	+35.77%	−24.53%
312 K	+12.92%	+37.04%	−24.12%
314 K	+17.77%	+42.03%	−24.26%

**Table 2:** Stern-Volmer constant K_SV_ [M^−1^] of the IBU-HSA system.

IBU-HSA	K_SV_ [M^−1^] ± SD [M^−1^] λex = 275 nm	K_SV_ [M^−1^] ± SD [M^−1^] λex = 295 nm	K_SV_ [M^−1^] ± SD [M^−1^] Tyr
308 K	2.83 × 10^4^ ± 0.38 × 10^3^	0.73 × 10^4^ ± 1.05 × 10^3^	0.23 × 10^4^ ± 1.24 × 10^3^
310 K	3.10 × 10^4^ ± 0.79 × 10^3^	0.88 × 10^4^ ± 0.54 × 10^3^	0.33 × 10^4^ ± 0.88 × 10^3^
312 K	3.95 × 10^4^ ± 1.12 × 10^3^	0.87 × 10^4^ ± 0.59 × 10^3^	0.38 × 10^4^ ± 0.55 × 10^3^
314 K	4.76 × 10^4^ ± 1.66 × 10^3^	1.26 × 10^4^ ± 0.36 × 10^3^	0.53 × 10^4^ ± 1.22 × 10^3^

SD: Standard deviation.

**Table 3:** Association constants K_a_ [M^−1^], mean number of IBU moles bound with one mole of HSA (*n*), and the Hill’s coefficient (*n_H_*) in the IBU-HSA system.

	Klotz Method		Hill Method	
λex = 275 nm	K_a_ [M^−1^] ± SD × 10^−4^ [M^−1^]	*n* ± SD	K_a_ [M^−1^] ± SD × 10^−4^ [M^−1^]	*n_H_* ± SD
308 K	0.51 ± 0.14	0.99 ± 0.03	0.11 ± 0.05	0.80 ± 0.12
310 K	1.14 ± 0.11	0.98 ± 0.07	0.87 ± 0.10	0.83 ± 0.10
312 K	1.02 ± 0.09	1.02 ± 0.05	0.79 ± 0.12	0.81 ± 0.11
314 K	2.59 ± 0.10	2.59 ± 0.08	2.57 ± 0.01	0.84 ± 0.08
	**Klotz Method**		**Hill Method**	
**λex = 295 nm**	**K_a_ [M^−1^] ± SD × 10^−4^ [M^−1^]**	***n* ± SD**	**K_a_ [M^−1^] ± SD × 10^−4^ [M^−1^]**	***n_H_* ± SD**
308 K	0.83 ± 0.09	2.02 ± 0.21	0.63 ± 0.04	0.84 ± 0.02
310 K	0.12 ± 0.03	1.01 ± 0.15	0.08 ± 0.02	0.89 ± 0.07
312 K	0.10 ± 0.05	1.01 ± 0.09	0.06 ± 0.02	0.81 ± 0.07
314 K	1.02 ± 0.14	0.98 ± 0.17	0.63 ± 0.04	0.92 ± 0.05
	**Klotz Method**		**Hill Method**	
**Tyr**	**K_a_ [M^−1^] ± SD × 10^−4^ [M^−1^]**	***n* ± SD**	**K_a_ [M^−1^] ± SD × 10^−4^ [M^−1^]**	***n_H_* ± SD**
308 K	0.09 ± 0.01	1.03 ± 0.03	0.10 ± 0.02	1.07 ± 0.05
310 K	0.34 ± 0.05	1.02 ± 0.05	0.34 ± 0.08	1.03 ± 0.05
312 K	0.06 ± 0.01	1.02 ± 0.03	0.07 ± 0.03	1.03 ± 0.05
314 K	0.51 ± 0.09	1.02 ± 0.02	0.57 ± 0.03	1.08 ± 0.07

SD: Standard deviation.

**Table 4:** Percent intensification (+)/quenching (−) of the IBU-HSA system fluorescence.

IBU-HSA		pH 6.5	pH 6.8	pH 7.4	pH 7.8	pH 8.1
308 K	Trp + Tyr λex = 275 nm	+0.70%	+1.39%	+10.57%	+4.58%	+8.32%
	Trp λex = 295 nm	+19.49%	+22.87%	+32.36%	+19.14%	+24.50%
	Tyr	−18.79%	−21.48%	−21.79%	−14.56%	−16.18%
310 K	Trp + Tyr λex = 275 nm	+0.04%	+1.41%	+11.24%	+4.40%	+8.15%
	Trp λex = 295 nm	+19.29%	+23.32%	+35.77%	+19.90%	+23.85%
	Tyr	−19.25%	−21.91%	−24.53%	−15.50%	−15.70%
312 K	Trp + Tyr λex = 275 nm	+ 2.86%	+3.60%	+12.92%	+4.84%	+9.14%
	Trp λex = 295 nm	+18.89%	+27.92%	+37.04%	+20.33%	+24.64%
	Tyr	−16.03%	−24.32%	−24.12%	−15.49%	−15.50%
314 K	Trp + Tyr λex = 275 nm	+10.60%	+7.19%	+17.77%	+5.93%	+8.69%
	Trp λex = 295 nm	+21.22%	+32.38%	+42.03%	+21.31%	+25.21%
	Tyr	−10.62%	−25.19%	−24.26%	−15.38%	−16.52%

**Table 5:** Stern-Volmer constants K_SV_ [M^−1^] calculated for IBU-HSA systems.

IBU-HSA		K_SV_ [M^−1^] ± SD × 10^−4^ [M^−1^]				
		pH 6.5	pH 6.8	pH 7.4	pH 7.8	pH 8.1
308 K	λex = 275 nm	0.36 ± 0.26	0.17 ± 0.06	2.83 ± 0.04	10.36 ± 1.96	18.85 ± 0.92
	λex = 295 nm	0.34 ± 0.11	0.10 ± 0.06	0.73 ± 0.11	0.38 ± 0.30	3.13 ± 0.02
	Tyr	1.49 ± 0.07	0.96 ± 0.19	0.23 ± 0.12	0.27 ± 0.08	2.17 ± 0.04
310 K	λex = 275 nm	0.78 ± 0.46	0.65 ± 0.08	3.10 ± 0.08	13.20 ± 7.48	26.26 ± 1.31
	λex = 295 nm	0.38 ± 0.08	0.26 ± 0.07	0.88 ± 0.05	1.45 ± 0.19	3.47 ± 0.02
	Tyr	1.36 ± 0.08	0.85 ± 0.14	0.33 ± 0.09	0.89 ± 0.04	1.53 ± 0.05
312 K	λex = 275 nm	0.99 ± 0.11	0.86 ± 0.08	3.95 ± 0.11	16.18 ± 10.56	30.95 ± 3.68
	λex = 295 nm	0.77 ± 0.03	0.33 ± 0.04	0.87 ± 0.06	2.67 ± 0.06	3.65 ± 0.01
	Tyr	1.38 ± 0.04	0.86 ± 0.14	0.38 ± 0.06	1.83 ± 0.08	1.45 ± 0.03
314 K	λex = 275 nm	1.06 ± 0.05	0.49 ± 0.18	4.76 ± 0.17	19.22 ± 18.48	36.43 ± 3.45
	λex = 295 nm	0.79 ± 0.07	0.37 ± 0.08	1.26 ± 0.04	2.93 ± 0.06	3.94 ± 0.03
	Tyr	0.36 ± 0.18	0.71 ± 0.05	0.53 ± 0.12	1.08 ± 0.04	1.79 ± 0.08

SD: Standard deviation.

**Table 6:** Association constants K_a_ [M^−1^] per the Klotz method, mean number of IBU moles bound with one mole of HSA (*n*) in the IBU-HSA system.

K_a_ [M^−1^] ± SD × 10^−4^ [M^−1^] *n* ± SD						
		pH 6.5	pH 6.8	pH 7.4	pH 7.8	pH 8.1
λex = 275 nm	308 K	2.41 ± 0.77	-	0.51 ± 0.14	7.65 ± 0.86	18.64 ± 1.61
		0.27 ± 0.15	-	0.99 ± 0.03	0.86 ± 0.02	0.82 ± 0.03
	310 K	5.35 ± 1.27	4.13 ± 0.77	1.14 ± 0.11	13.88 ± 3.57	13.98 ± 0.93
		0.07 ± 0.03	1.80 ± 0.28	0.98 ± 0.07	0.92 ± 0.05	0.82 ± 0.03
	312 K	2.95 ± 1.72	1.41 ± 0.19	1.02 ± 0.09	15.72 ± 3.26	11.22 ± 0.85
		0.22 ± 0.08	2.72 ± 0.32	1.02 ± 0.05	0.93 ± 0.04	0.97 ± 0.07
	314 K	0.57 ± 0.10	0.38 ± 0.14	2.59 ± 0.10	17.34 ± 4.65	3.91 ± 0.01
		0.14 ± 0.03	2.39 ± 0.27	2.59 ± 0.08	0.93 ± 0.04	0.96 ± 0.01
λex = 295 nm	308 K	0.37 ± 0.08	0.58 ± 0.20	0.83 ± 0.09	2.03 ± 0.06	3.91 ± 0.01
		0.99 ± 0.25	1.05 ± 0.36	2.02 ± 0.21	0.32 ± 0.02	0.96 ± 0.01
	310 K	0.37 ± 0.06	0.48 ± 0.29	0.12 ± 0.03	2.27 ± 0.12	3.14 ± 0.03
		0.99 ± 0.17	1.03 ± 0.20	1.01 ± 0.15	0.81 ± 0.08	0.96 ± 0.03
	312 K	0.82 ± 0.03	0.09 ± 0.13	0.10 ± 0.05	3.11 ± 0.06	2.92 ± 0.05
		0.98 ± 0.04	1.00 ± 0.23	1.01 ± 0.09	0.96 ± 0.05	0.96 ± 0.05
	314 K	0.84 ± 0.07	0.26 ± 0.18	1.02 ± 0.14	3.45 ± 0.05	4.40 ± 0.00
		0.98 ± 0.10	1.01 ± 0.22	0.98 ± 0.17	0.96 ± 0.05	0.96 ± 0.00
Tyr	308 K	2.09 ± 0.12	1.04 ± 0.20	0.09 ± 0.01	0.04 ± 0.14	3.25 ± 0.07
		0.96 ± 0.10	0.97 ± 0.26	1.03 ± 0.03	1.04 ± 1.13	1.15 ± 0.07
	310 K	1.21 ± 0.09	0.43 ± 0.09	0.34 ± 0.05	0.58 ± 0.11	1.70 ± 0.06
		0.97 ± 0.10	0.98 ± 0.26	1.02 ± 0.05	0.98 ± 0.23	0.96 ± 0.05
	312 K	1.89 ± 0.08	0.93 ± 0.14	0.06 ± 0.01	2.08 ± 0.09	1.61 ± 0.04
		0.97 ±0.07	0.97 ± 0.20	1.02 ± 0.03	0.96 ± 0.07	0.97 ± 0.04
	314 K	1.16 ± 0.45	1.06 ± 0.11	0.51 ± 0.09	1.17 ± 0.04	2.03 ± 0.09
		1.21 ± 0.22	0.97 ± 0.13	1.02 ± 0.02	0.97 ± 0.05	0.96 ± 0.08

SD: Standard deviation.

**Table 7:** Association constants K_a_ [M^−1^] per the Hill method and the Hill’s coefficient (*n_H_*) in the IBU-HSA system.

K_a_ [M^−1^] ± SD × 10^−4^ [M^−1^] *n_H_* ± SD						
		pH 6.5	pH 6.8	pH 7.4	pH 7.8	pH 8.1
λex = 275 nm	308 K	1.75 ± 0.34	0.70 ± 0.05	0.11 ± 0.05	13.37 ± 4.67	0.81 ± 0.17
		0.61 ± 0.17	2.98 ± 0.35	0.80 ± 0.12	1.60 ± 0.27	0.46 ± 0.14
	310 K	0.73 ± 0.18	3.51 ± 0.33	0.87 ± 0.10	7.41 ± 1.81	0.13 ± 0.04
		2.15 ± 0.08	3.14 ± 0.57	0.83 ± 0.10	1.94 ± 0.34	0.45 ± 0.05
	312 K	6.01 ± 1.46	2.41 ± 0.04	0.79 ± 0.12	5.57 ± 0.61	0.10 ± 0.02
		1.03 ± 0.07	1.76 ± 0.13	0.81 ± 0.11	2.35 ± 0.25	0.36 ± 0.02
	314 K	0.35 ± 0.08	1.38 ± 0.03	2.57 ± 0.01	7.07 ± 1.44	0.05 ± 0.02
		0.87 ± 0.10	1.54 ± 0.10	0.84 ± 0.08	2.24 ± 0.34	0.42 ± 0.08
λex = 295 nm	308 K	0.32 ± 0.02	2.33 ± 0.29	0.63 ± 0.04	0.14 ± 0.03	3.54 ± 0.03
		0.95 ± 0.03	1.93 ± 0.03	0.85 ± 0.04	0.65 ± 0.05	0.97 ± 0.02
	310 K	0.33 ± 0.02	1.40 ± 0.25	0.08 ± 0.02	1.28 ± 0.07	2.84 ± 0.01
		0.96 ± 0.03	1.44 ± 0.03	0.89 ± 0.07	0.75 ± 0.06	0.92 ± 0.03
	312 K	0.75 ± 0.03	0.90 ± 0.10	0.06 ± 0.02	2.93 ± 0.02	2.62 ± 0.01
		0.94 ± 0.04	0.99 ± 0.04	0.81 ± 0.07	0.81 ± 0.03	0.90 ± 0.04
	314 K	0.77 ± 0.02	0.44 ± 0.09	0.63 ± 0.04	3.28 ± 0.04	4.00 ± 0.03
		0.98 ± 0.02	1.11 ± 0.04	0.92 ± 0.05	0.81 ± 0.04	0.94 ± 0.01
Tyr	308 K	2.09 ± 0.02	1.14 ± 0.05	0.10 ± 0.02	0.03 ± 0.00	4.70 ± 0.47
		1.11 ± 0.07	1.11 ± 0.06	1.07 ± 0.05	0.91 ± 0.06	1.45 ± 0.23
	310 K	1.22 ± 0.03	0.44 ± 0.03	0.34 ± 0.08	0.50 ± 0.03	1.64 ± 0.02
		1.05 ± 0.03	1.02 ± 0.04	1.03 ± 0.05	0.93 ± 0.04	1.03 ± 0.03
	312 K	1.84 ± 0.02	0.99 ± 0.04	0.07 ± 0.03	1.99 ± 0.01	1.54 ± 0.01
		1.04 ± 0.04	1.08 ± 0.05	1.03 ± 0.05	1.04 ± 0.03	1.01 ± 0.02
	314 K	2.59 ± 0.04	1.09 ± 0.03	0.57 ± 0.03	1.08 ± 0.01	1.96 ± 0.02
		1.96 ± 0.42	1.06 ± 0.04	1.08 ± 0.07	0.96 ± 0.02	1.06 ± 0.04

SD: Standard deviation.

## Supplementary Materials

The following are available online at https://www.mdpi.com/1424-8247/13/9/205/s1, Figure S1: (**a**) The spectrophotometric spectra; (**b**) second derivative absorption spectra of human serum albumin (5 × 10^−6^ M), T = 308–314 K, pH = 7.4.; Figure S2: (**a**,**c**,**e**,**g**) The spectrophotometric spectra; (**b**,**d**,**f**,**h**) second derivative absorption spectra of human serum albumin (5 × 10^−6^ M) at various concentrations of ibuprofen (1 × 10^−5^–1 × 10^−4^ M) in temperature (**a**,**b**) T = 308 K; (**c**,**d**) T = 310 K; (**e**,**f**) T = 312 K; (**g**,**h**) T = 314 K, pH = 7.4.; Figure S3: The fluorescence quenching spectra of human serum albumin (5 × 10^−6^ M) at various concentrations of ibuprofen (1 × 10^−5^–1 × 10^−4^ M); (**a**) T = 308 K; (**b**) T = 310 K; (**c**) T = 312 K; (**d**) T = 314 K, λex = 275 nm and λex = 295 nm (in the insert), pH = 7.4., Figure S4: Fluorescence intensification/quenching curves of human serum albumin (5 × 10^−6^ M):tryptophan+tyrosine (■); tryptophan (●); tyrosine (▲), at various concentrations of ibuprofen (1 × 10^−5^–1 × 10^−4^ M) in (**a**) T = 308 K; (**b**) T = 310 K; (**c**) T = 312 K; (**d**) T = 314 K, pH = 7.4., Figure S5: The Stern-Volmer curves modified by Lehrer for the binary systems IBU-HSA complex in T = 308 K (■); T = 310 K (●); T = 312 K (▲); T = 314 K (◆), (**a**) λex = 275 nm − tryptophan+tyrosine, (**b**) λex = 295 nm − tryptophan, (**c**) differential spectrum − tyrosine, pH = 7.4., Figure S6: Scatchard curves of r/([L_f_] vs. r(molar ratio L_b_: HSA) for for the binary systems IBU-HSA complex in T = 308 K (■); T = 310 K (●); T = 312 K (▲); T = 314 K (◆), (**a**) λex = 275 nm − tryptophan+tyrosine, (**b**) λex = 295 nm − tryptophan, (**c**) differential spectrum − tyrosine, pH = 7.4., Figure S7: Klotz curves of 1/r vs. 1/([L_f_] for the binary systems IBU-HSA complex in T = 308 K (■); T = 310 K (●); T = 312 K (▲); T = 314 K (◆), (**a**) λex = 275 nm − tryptophan+tyrosine, (**b**) λex = 295nm − tryptophan, (**c**) differential spectrum − tyrosine, pH = 7.4., Figure S8: Hill curves of log (r/(1 − r)) vs. log[L_f_] for the binary systems IBU-HSA complex in T = 308 K (■); T = 310 K (●); T = 312 K (▲); T = 314 K (◆), (**a**) λex = 275 nm − tryptophan + tyrosine, (**b**) λex = 295nm − tryptophan, (**c**) differential spectrum − tyrosine, pH = 7.4., Figure S9: The second derivative absorption spectra of human serum albumin (5 × 10^−6^ M) at various concentrations of ibuprofen (1 × 10^−5^–1 × 10^−4^ M) in temperature T = 308 K (**‒**); T = 310 K (**‒**); T = 312 K (**‒**); T = 314 K (**‒**) for (**a**) pH 6.5; (**b**) pH 6.8; (**c**) pH 7.4; (**d**) pH 7.8; (**e**) pH 8.1.
