# Supplementary material for: A Comprehensive Spectroscopic Analysis of the Ibuprofen Binding with Human Serum Albumin, Part I
Source: Pharmaceuticals (Basel). 2020 Aug 21;13(9):205. doi: 10.3390/ph13090205 (PMC7557384; doi:10.3390/ph13090205)
Supplement: Supplementary file 1 [file pharmaceuticals-13-00205-s001.zip › Supplementary caption.docx]

**Supplementary Figure S1.** (**a**) The spectrophotometric spectra; (**b**) second derivative absorption spectra of human serum albumin (5 × 10^−6^ M), T = 308 ÷ 314 K, pH = 7.4.

**Supplementary Figure S2.** (**a**, **c**, **e**, **g**) The spectrophotometric spectra; (**b**, **d**, **f**, **h**) second derivative absorption spectra of human serum albumin (5 × 10^−6^ M) at various concentrations of ibuprofen (1 × 10^−5^ ÷ 1 × 10^−4^ M) in temperature (**a, b**) T = 308 K; (**c, d**) T = 310 K; (**e, f**) T = 312 K; (**g, h**) T = 314 K, pH = 7.4.

**Supplementary Figure S3.** The fluorescence quenching spectra of human serum albumin (5 × 10^−6^ M) at various concentrations of ibuprofen (1 × 10^−5^ ÷1 × 10^−4^ M); (**a**) T = 308 K; (**b**) T = 310 K; (**c**) T = 312 K; (**d**) T = 314 K, λex = 275 nm and λex = 295 nm (in the insert), pH = 7.4.

**Supplementary Figure S4**. Fluorescence intensification/quenching curves of human serum albumin (5 × 10^−6^ M):tryptophan+tyrosine (■); tryptophan (●); tyrosine (▲), at various concentrations of ibuprofen (1 × 10^−5^ ÷ 1 × 10^−4^ M) in (**a**) T = 308 K; (**b**) T = 310 K; (**c**) T = 312 K; (**d**) T = 314 K, pH = 7.4.

**Supplementary Figure S5.** The Stern-Volmer curves modified by Lehrer for the binary systems IBU-HSA complex in T = 308 K (■); T = 310 K (●); T = 312 K (▲); T = 314 K (◆), (**a**) λex = 275 nm - tryptophan+tyrosine, (**b**) λex = 295 nm - tryptophan, (c) differential spectrum - tyrosine, pH = 7.4.

**Supplementary Figure S6**. Scatchard curves of r/([L_f_] vs r(molar ratio L_b_: HSA) for for the binary systems IBU-HSA complex in T = 308 K (■); T = 310 K (●); T = 312 K (▲); T = 314 K (◆), (**a**) λex = 275 nm - tryptophan+tyrosine, (**b**) λex = 295 nm - tryptophan, (**c**) differential spectrum - tyrosine, pH = 7.4.

**Supplementary Figure S7**. Klotz curves of 1/r vs 1/([L_f_] for the binary systems IBU-HSA complex in T = 308 K (■); T = 310 K (●); T = 312 K (▲); T = 314 K (◆), (**a**) λex = 275 nm - tryptophan+tyrosine, (**b**) λex = 295nm - tryptophan, (**c**) differential spectrum - tyrosine, pH = 7.4.

**Supplementary Figure S8**. Hill curves of log (r/(1 − r)) vs log[L_f_] for the binary systems IBU-HSA complex in T = 308 K (■); T = 310 K (●); T = 312 K (▲); T = 314 K (◆), (**a**) λex = 275 nm – tryptophan + tyrosine, (**b**) λex = 295nm - tryptophan, (**c**) differential spectrum - tyrosine, pH =7.4.

**Supplementary Figure S9.** The second derivative absorption spectra of human serum albumin (5 × 10^−6^ M) at various concentrations of ibuprofen (1 × 10^−5^ ÷ 1 × 10^−4^ M) in temperature T = 308 K (**‒**); T = 310 K (**‒**); T = 312 K (**‒**); T = 314 K (**‒**) for (**a**) pH 6.5; (**b**) pH 6.8; (**c**) pH 7.4; (**d**) pH 7.8; (**e**) pH 8.1.
