# Supplementary material for: A Comprehensive Spectroscopic Analysis of the Ibuprofen Binding with Human Serum Albumin, Part I
Source: Pharmaceuticals (Basel). 2020 Aug 21;13(9):205. doi: 10.3390/ph13090205 (PMC7557384; doi:10.3390/ph13090205)
Supplement: Supplementary file 1 [file pharmaceuticals-13-00205-s001.zip › Supplementary Figure S1.docx]

|  |  |
| --- | --- |

**Supplementary Figure S1.** (**a**) The spectrophotometric spectra; (**b**) second derivative absorption spectra of human serum albumin (5 × 10^−6^ M), T = 308 ÷ 314 K, pH = 7.4.
