# Supplementary material for: A Comprehensive Spectroscopic Analysis of the Ibuprofen Binding with Human Serum Albumin, Part I
Source: Pharmaceuticals (Basel). 2020 Aug 21;13(9):205. doi: 10.3390/ph13090205 (PMC7557384; doi:10.3390/ph13090205)
Supplement: Supplementary file 1 [file pharmaceuticals-13-00205-s001.zip › Supplementary Figure S9.docx]

|  |  |
| --- | --- |
|  |  |
|  | |

**Supplementary Figure S9.** The second derivative absorption spectra of human serum albumin
(5 × 10^−6^ M) at various concentrations of ibuprofen (1 × 10^−5^ ÷ 1 × 10^−4^ M) in temperature T = 308 K (**‒**);
T = 310 K (**‒**); T = 312 K (**‒**); T = 314 K (**‒**) for (**a**) pH 6.5; (**b**) pH 6.8; (**c**) pH 7.4; (**d**) pH 7.8; (**e**) pH 8.1.
