# Supplementary material for: A Comprehensive Spectroscopic Analysis of the Ibuprofen Binding with Human Serum Albumin, Part I
Source: Pharmaceuticals (Basel). 2020 Aug 21;13(9):205. doi: 10.3390/ph13090205 (PMC7557384; doi:10.3390/ph13090205)
Supplement: Supplementary file 1 [file pharmaceuticals-13-00205-s001.zip › Supplementary Figure S3.doc]

|  |  |
| --- | --- |
|  |  |

**Supplementary Figure S3.** The fluorescence quenching spectra of human serum albumin (5 × 10^−6^ M) at various concentrations of ibuprofen (1 × 10^−5^ ÷1 × 10^−4^ M); (**a**) T = 308 K; (**b**) T = 310 K; (**c**) T = 312 K; (**d**) T = 314 K, λex = 275 nm and λex = 295 nm (in the insert), pH = 7.4.
