# Supplementary material for: A Comprehensive Spectroscopic Analysis of the Ibuprofen Binding with Human Serum Albumin, Part I
Source: Pharmaceuticals (Basel). 2020 Aug 21;13(9):205. doi: 10.3390/ph13090205 (PMC7557384; doi:10.3390/ph13090205)
Supplement: Supplementary file 1 [file pharmaceuticals-13-00205-s001.zip › Supplementary Figure S6.doc]

|  |  |
| --- | --- |
|  | |

**Supplementary Figure S6**. Scatchard curves of r/([L_f_] vs r(molar ratio L_b_: HSA) for for the binary systems IBU-HSA complex in T = 308 K (■); T = 310 K (●); T = 312 K (▲); T = 314 K (◆), (**a**) λex = 275 nm - tryptophan+tyrosine, (**b**) λex = 295 nm - tryptophan, (**c**) differential spectrum - tyrosine, pH = 7.4.
